# Accuracy of Two-Dimensional Transvaginal Sonography and Office Hysteroscopy for Detection of Uterine Abnormalities in Patients with Repeated Implantation Failures or Recurrent Pregnancy Loss

**DOI:** 10.22074/ijfs.2018.5034

**Published:** 2017-10-14

**Authors:** Marzieh Shiva, Firouzeh Ahmadi, Arezoo Arabipoor, Mansoureh Oromiehchi, Mohammad Chehrazi

**Affiliations:** 1 Department of Endocrinology and Female Infertility, Reproductive Biomedicine Research Center, Royan Institute for Reproductive Biomedicine, ACECR, Tehran, Iran; 2 Department of Reproductive Imaging, Reproductive Biomedicine Research Center, Royan Institute for Reproductive Biomedicine, ACECR, Tehran, Iran; 3 Department of Epidemiology and Reproductive Health, Reproductive Epidemiology Research Center, Royan Institute for Reproductive Biomedicine, ACECR, Tehran, Iran

**Keywords:** Diagnosis, Hysteroscopy, Ultrasonography, Uterine

## Abstract

**Background:**

We sought to compare diagnostic values of two-dimensional transvaginal sonography (2D TVS) and office hysteroscopy (OH) for evaluation of endometrial pathologies in cases with repeated implantation failure (RIF) or recurrent pregnancy loss (RPL).

**Materials and Methods:**

This prospective study was performed at Royan Institute from December 2013 to January 2015. TVS was performed before hysteroscopy as part of the routine diagnostic work-up in 789 patients with RIF or RPL. Uterine biopsy was performed in cases with abnormal diagnosis in TVS and/or hysteroscopy. We compared the diagnostic accuracy values of TVS in detection of uterine abnormalities with OH by receiver operating characteristic (ROC) curve analysis.

**Results:**

TVS examination detected 545 (69%) normal cases and 244 (31%) pathologic cases, which included 84 (10.6%) endometrial polyps, 15 (1.6%) uterine fibroids, 10 (1.3%) Asherman’s syndrome, 9 (1.1%) endometrial hy- pertrophy, and 126 (15.9%) septate and arcuate uterus. TVS and OH concurred in 163 pathologic cases, although TVS did not detect some pathology cases (n=120). OH had 94% sensitivity, 95% specificity, 62% positive predictive value (PPV), and 99% negative predictive value (NPV) for detection of endometrial polyps. In the diagnosis of myoma, sen- sitivity, specificity, PPV, and NPV were 100%. TVS had a sensitivity of 50% and specificity of 98% for the diagnosis of myoma. For polyps, TVS had a sensitivity of 54% and specificity of 80%. Area under the ROC curve (AUROC) was 70.69% for the accuracy of TVS compared to OH.

**Conclusion:**

TVS had high specificity and low sensitivity for detection of uterine pathologies in patients with RIF or RPL compared with OH. OH should be considered as a workup method prior to treatment in patients with normal TVS findings.

## Introduction

Intrauterine pathologies are present in 25-50% of infertile patients (1). Structural abnormalities of the uterine endometrial cavity affect reproduction outcomes because they interfere with implantation or cause spontaneous abortions (2). Therefore, accurate diagnosis of any endometrial pathology in the patient is an important step prior to beginning the assisted reproductive technology (ART) cycles (2,3). During the last decades, hysterosalpingography (HSG), hysteroscopy, sonohysterography, and transvaginal sonography (TVS) have been developed to evaluate the uterine cavity; each has their own advantages and disadvantages (4,7). TVS is universally considered the initial, non-invasive procedure for assessment of intrauterine pathologies (8). Hysteroscopy allows for direct and three-dimensional (3D) visualization, and sampling of the uterine cavity. Although considered the gold standard (9), it is not as affordable and comfortable as TVS, which has relatively lower patient discomfort (4). Since the introduction of the hysteroscopic technique, the procedure has undergone considerable modifications, leading to an increase in patient compliance and tolerance. Fiberoptics, smaller caliber of the endoscopes, use of simpler distention media, and availability of safer local infiltrative anesthetics have all contributed to the increased use of this technique to evaluate the uterine cavity in the office setting (10,11). Diagnostic or office hysteroscopy (OH), though increasingly used for uterine cavity evaluation, is still underutilized (11). Some studies have evaluated the diagnostic values of two-dimensional (2D) and/or 3D TVS compared with hysteroscopy (1,4,8,12). El-Mazny et al. (1) compared TVS and OH for evaluation of intrauterine pathologies in patients scheduled for ART. They have concluded that the TVS was specific (100%), but not sensitive (41.7%) compared to OH. However, the value of OH as a routine evaluation in the management of infertile women is a debatable topic. The goal of this prospective study was to evaluate the diagnostic validity of 2D TVS and outpatient OH in the detection of uterine cavity pathologies in recurrent implantation failure (RIF) or recurrent abortion cases. 

## Materials and Methods

We conducted this prospective cohort study at Royan Institute between December 2013 and January 2015. The Institution Review Board and the Ethics Committee of Royan Institute approved this study. All patients signed a consent form to give permission to use their treatment outcomes confidentially without mentioning the name. All TVS evaluations were performed free for participants. We included all patients with primary and secondary infertility, 20 to 40 years of age, who were diagnosed with failed *in vitro* fertilization (IVF), intrauterine insemination (IUI), or recurrent pregnancy loss (RPL). Exclusion criteria were as follows: history of previous surgery and pathology in the uterus, or patients with heterogenic or echogenic endometrium attributed to bleeding. 

RIF was defined as the lack of clinical pregnancy after transfer of at least four good-quality embryos in a minimum of three fresh or frozen cycles in women under the age of 40 years (13). RPL referred to two or more failed clinical pregnancies as recorded by ultrasonography or histopathologic evaluations in infertile women. During the study period, we initially evaluated all eligible patients by TVS, then OH in the same month. Uterine biopsy was performed in cases with abnormal diagnosis according to TVS and/or hysteroscopy. For example, in complex situations like submucous myoma, endometrial polyps and extensive Asherman’s syndrome we scheduled several hysteroscopic procedures and performed therapeutic interventions after obtaining informed consent from the patients. 

### Uterine assessment

Patients underwent TVS in the follicular phase of the menstrual cycle (days 5-13) when the menstrual bleeding stopped and before the diagnostic hysteroscopy evaluation. TVS and OH were carried out in the same cycle. All sonographic evaluations were performed by an expert radiologist (F.A.) using an Aloka α-10-color doppler with a transvaginal 6 MHz probe. Uterine cavity abnormalities that included polyp lesions, uterine myoma, septate and arcuate uterus, adhesion, and endometrial hypertrophy were evaluated. We have defined a polyp as a round or oval echogenic lesion with intact endometrial-myometrial junction located in the endometrial cavity. Submucosal fibroma is a benign lesion that originates from the smooth muscle layer and the accompanying connective tissue of the uterus. It is observed in sonography as a mixed or hypoechoic mass lesion that originates from the myometrium and interrupts the endometrial layer. Septum is a form of congenital malformation that divides the uterine cavity by a longitudinal short or long wall whereas the outside of the uterus has a normal shape. Abnormal adhesion is detected as an irregular endometrial line in ultrasound and observed as a fibrous band which separates the endometrial cavity. Endometrial hypertrophy is detected as thickening of the endometrium on sonography which represents excessive proliferation of the endometrium cells (14). 

An expert gynecologist performed the OH the next day by using a rigid hysteroscope (Oblique Telescope 30°, diameter: 2 mm, length: 26 cm, KARL STORZ GmbH & Co., Germany) assembled in a 4.2 mm diameter diagnostic sheath with an atraumatic tip. A high-intensity cold light source and fiberoptic cable were used to clarify the uterine cavity. Normal saline (0.9%) was applied as the distention medium, with pressure maintained between 100-120 mm Hg using a pressure adjustable cuff system to achieve the lowest adequate pressure to distend the uterine cavity. This practically painless procedure does not require the use of analgesics or sedatives. 

The patient was placed in the dorsolithotomy position and a pelvic examination was performed to detect the size of the uterus and its direction. No speculum or tenaculum were needed as the vaginoscopic ‘‘no touch’’ technique was applied. Once this was accomplished, the hysteroscope with its light source was placed at the level of the ectocervix, and guided into the endocervical canal. At the entrance of uterine cavity, a systematic observation was performed that included the uterine cornua, tubal ostia, uterine fundus, lateral, anterior, and posterior uterine walls. The uterine cavity and endocervical canal were reevaluated during withdrawal of the instrument. 

A video system was used for patient observation and to document the procedure for future reference. The patients were under observation for a minimum of 30 minutes to assess for possible side effects and complications. The gynecologist who performed the diagnostic hysteroscopy was unaware of the TVS results in order to minimize performance bias. OH were recorded on a special data form that included the following items: i. Appearance and figure of the endocervical canal (endocervicitis-determined by congestion and hypertrophy of the mucosal lining; mucous polyp-associated with contact bleeding and excessive discharge), ii. Endometrial appearance (endometritis-congestion, hyperemia, hemorrhages, and adhesions; hyperplastic endometrium-thickened and easily indented by pressure, with or without multiple polyps), iii. Figure of the uterine cavity, and iv. Existence and situation of structural lesions (myomas, polyps, adhesions, and congenital anomalies). 

### Statistical analysis

All data were recorded in the Statistical Package for the Social Sciences (SPSS) version 20 (SPSS Inc., Chicago, IL, USA) and analyzed using appropriate accuracy indices. Diagnostic accuracy was assessed by sensitivity, specificity, positive and negative predictive values (PPV, NPV) calculated using 2×2 tables for each method. The agreement between the two methods for the detection of uterine lesions was calculated by the Kappa coefficient. Histopathologic results were the gold standard. Cases with abnormal OH had only a uterine pathologic assessment. Because the results of the sonography test affect whether the gold standard (pathology) procedure is used to verify the test result, verification bias exists when sonography results are compare with pathology findings. We have used previous methods (15,17) to adjust the verification bias. The McNemar test was used to compare marginal homogeneity for results of 2D TVS and OH. Diagnostic accuracy value for results of the 2D TVS compared OH findings was calculated through the receiver operating characteristic (ROC) curve. 

## Results

All planned procedures were completed successfully. There were no complications recorded during or after the procedures. During the study period, we evaluated 789 patients with both 2D TVS and OH. In the TVS examination, 545 (69%) cases were normal and 244 (31%) had pathologic findings that included 84 (10.6%) polyp lesions, 15 (1.6%) uterine fibroids, 10 (1.3%) Asherman’s syndrome, 9 (1.1%) endometrial hypertrophy, and 126 (15.9%) septate and arcuate uterus. TVS and OH results were in agreement in 163 pathologic cases. However TVS could not detect some pathology (n=120, Table 1). The McNemar test showed that the diagnostic values for detection of Asherman’s syndrome, endometrial hypertrophy, arcuate and septate uterus significantly differed between the two methods (Table 1). 

**Fig.1:**
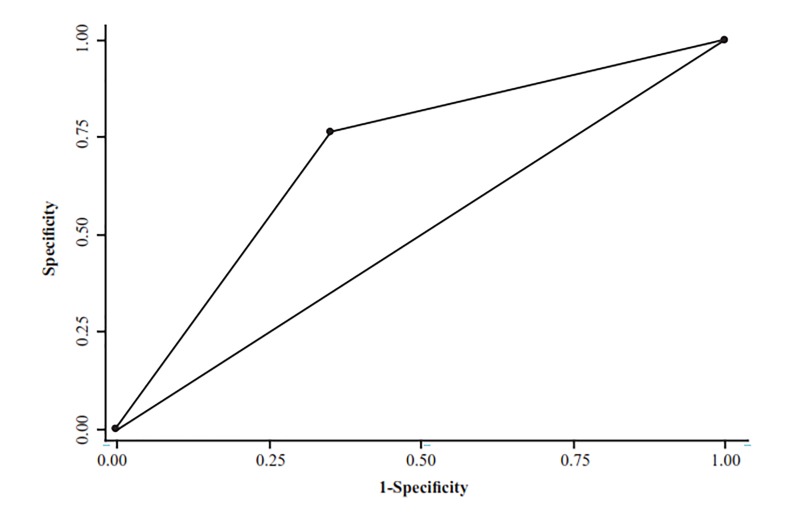
Area under ROC curve (AUC=0.70) for accuracy of two dimensional trans-vaginal sonography in compare to office hysteroscopy.

Area under ROC curve (AUROC) was almost acceptable 70.69% for the accuracy of 2D TVS compared with OH (Fig .1). 

## Polyp lesions

There was a significant agreement between TVS and OH in the diagnosis of uterine polyps. The Kappa coefficient of 0.5 indicated an intermediate match. The results showed that TVS had 54% sensitivity and 80% specificity to detect polyps, with a reported PPV of 19% and NPV of 95% (Table 1). 

## Uterine fibroids

The results demonstrated a sensitivity of 50% and specificity of 98% for TVS to diagnosis myoma, with a PPV of 20% and NPV of 99%. There was a significant agreement between TVS and OH in the diagnosis of uterine fibroids. The Kappa coefficient was 0.3 and this agreement level was weak. 

**Table 1:** Total finding by TVS and OH in 789 patients


	2D TVSn (%)	OHn (%)	SN (%)	SP (%)	PPV (%)	NPV (%)	Kappa (%)	P value^a^

Normal finding	545 (69)	506 (64.1)						
Polyps	84 (10.6)	97 (12.3)	54	80	19	95	50	0.18
Submucosal myoma	15 (1.6)	15 (1.9)	50	98	20	99	30	1.00
Asherman’s syndrome	10 (1.3)	23 (2.9)	30	97.4	13	96.2	16	0.02
Endometrial hyperplasia	9 (1.1)	19 (2.4)	66.7	98.2	31.6	99.6	42	0.02
Arcuate uterus	87 (11)	65 (8.2)	29.9	94.4	40	91.6	27	0.03
Septate uterus	39 (4.9)	64 (8.1)	61.5	94.7	37	97.9	45	0.01
Total pathologic findings	244 (31)	283 (35.9)						


2D TVS; Two-dimensional transvaginal sonography, OH; Office hysteroscopy, SN; Sensitivity, SP; Specificity, PPV; Positive predictive value, NPV; Negative predictive value, and a; P value was computed using the McNemar test statistic.

## Endometrial hypertrophy

TVS and OH provided consistent results for the diagnosis of endometrial hypertrophy. The Kappa coefficient was 0.42 and this agreement level was intermediate. The results revealed that a sensitivity of 66.7% and specificity of 98.2% for TVS to diagnose endometrial hypertrophy. TVS had a PPV of 31.6% and NPV of 99.6%. 

## Asherman’s syndrome

There was a significant agreement between TVS and OH in the diagnosis of Asherman’s syndrome. The Kappa coefficient was 0.16; however, this matching level was weak. The analysis showed a sensitivity of 30% and specificity of 97.4% for TVS to diagnose Asherman’s syndrome. TVS had a calculated PPV of 13% and NPV of 96.2%. 

## Septate and arcuate uterus

There was significant agreement between TVS and OH in the diagnosis of septate uterus. The consistent level was intermediate with an obtained Kappa coefficient of 0.45. The results demonstrated a sensitivity of 61.5% and specificity of 94.7% for TVS to diagnose septate uterus. TVS had a PPV of 37.5% and NPV of 97.9%. The agreement between TVS and OH to diagnose arcuate uterus was significant. The consistent level was weak with a Kappa coefficient of 0.27. We obtained a sensitivity of 29.9% and specificity of 94.4% for TVS to diagnose arcuate uterus. The PPV and NPV were calculated as 40 and 91.6%.

## Discussion

Different types of uterine lesions (polys, fibroma, congenital anomalies and acquired disease) can play an important role in female reproductive failures. Various methods are used to diagnose these uterine pathologies. Hysteroscopy is an endoscopic evaluation of the uterine cavity with video recording capabilities, which enables a second opinion (1). This test can be performed in a clinic office without the need for anesthesia (18). Direct visual imaging of the uterine cavity by this method allows for the diagnosis of cancer, as well as polyps and submucosal myomas (19). Diagnostic hysteroscopy is considered a gold standard method for evaluation of the uterine cavity, with the capability for uterine pathology treatment particularly for women with RIF and RPL, as well as other infertile women (13,20,21). TVS is a noninvasive technique to evaluate the uterine cavity (14). The present study aims to evaluate the diagnostic value of TVS performed preceding to routine hysteroscopy to determine if TVS can alleviate the number of diagnostic hysteroscopies commonly performed in women with normal uterine cavities. 

Our results demonstrated the following: sensitivity (54%), specificity (80%), PPV (19%), and NPV (95%) of TVS for the diagnosis of endometrial polyps. These results for detection of myoma were: sensitivity (50%), specificity (98%), PPV (20%), and NPV (99%). Our results agreed with a number of studies (1,4,12,22). Babacan et al. (4) reported that TVS has a sensitivity of 54% and specificity of 84% for detecting endometrial polyps. Cepni et al. (12) found that TVS had a sensitivity of 58% and specificity of 94% for detection of intra-cavity fibromas. These researchers determined that TVS had a sensitivity of 72% and specificity of 50% for diagnosis of endometrial polyps. Bonnamy et al. (22) reported that TVS had a sensitivity of 57% and specificity of 69% for detection of intrauterine masses. Wanderley et al. (23), observed that TVS has a diagnostic accuracy of 65.9% for polyps, 78.1% for myoma, and 63.2% for endometrial hyperplasia. However, other studies reported different results (5,6,24). 

Niknejadi et al. (14) reported that TVS had sensitivity of 89.2% and specificity of 99.6% for endometrial fibroids. Soares et al. (24) and Loverro et al. (6) demonstrated that TVS had a sensitivity of 75-85% and specificity of 90-100% for the diagnosis of endometrial polyps. Balić and Balić (25), in a retrospective study, reported that TVS and hysteroscopy had identical sensitivity (100%) for diagnosis of endometrial polyps, whereas hysteroscopy had higher specificity (92.3%) than TVS (56.4%). The authors concluded that the agreement between hysteroscopy and histology was good, whereas there was moderate agreement between TVS and histology. On other hand, Krampl et al. (26) stated that TVS had a sensitivity of 23% and specificity of 93% for the diagnosis of intracavitary lesions in patients with abnormal uterine bleeding. The TVS sensitivity in their study was less than other studies. Fedele et al. (27) found that TVS had a misdiagnosis rate of 4.2% and was less effective than hysteroscopy for detection of polyps; however, they reported a sensitivity of 91% and specificity of 100% in TVS for detection of uterine adhesions. They concluded that TVS was a noninvasive, relatively inexpensive, possibly effective method to screen for uterine adhesions in high risk population (27). 

In the present study, we demonstrated that OH had a sensitivity of 94% and specificity of 95% for detection of endometrial polyps. Both values were 100% for diagnosis of myoma. Grimbizis et al. (8) showed that OH had a sensitivity of 97% and specificity of 91% for diagnosis of endometrial polyps, as well as a sensitivity of 100% and specificity of 98% for detection of myoma. Niknejadi et al. (9) reported excellent specificity (91.2%), good sensitivity (88.2%), an 81.4% PPV, and a 94.6% NPV for TVS in detection of uterine polyps. 

Our finding presented that 2D TVS has a low sensitivity and high specificity for diagnosis of septate and arcuate uterus. Recently, some studies evaluated the accuracy of 3D TVS for diagnosis of uterine anomalies (28,34). Szkodziak et al. (34) concluded that HSG was not an optimal procedure to diagnose uterine anomalies, whereas 3D TV USG could precisely determine uterus anomalies and might be considered as alternative to MRI. Ludwin et al. (33) reported that 3D-SIS was the same as hysteroscopy performed in conjunction with laparoscopy (HL) with the highest accuracy and also there was no significant difference in diagnostic value between 3D TVS with 2D SIS and 3D SIS or between expert 2D TVS and 3D TVS with 2D SIS. Despite the high diagnostic value of these ultrasound devices, is endoscopy necessary for differential diagnosis of common uterine abnormalities? The Thessaloniki ESHRE/ESGE consensus have recommended 3D-TVS for the diagnosis of female genital anomalies in high-risk, symptomatic women and in any asymptomatic patient suspected of having an anomaly discovered during a routine workup. This consensus suggested that the different diagnostic tools should be applied in an accurate manner and performed by experts to prevent mis-, overand underdiagnoses. The role of a combined ultrasound evaluation and OH should be prospectively examined in future researches (35). 

In the present study, we did not obtain endometrial biopsies for histologic evaluation in patients who had normal hysteroscopy and 2D TVS. However, we used statistical methods to adjust for verification bias to determine a diagnostic value of 2D TVS for endometrial abnormalities. 

## Conclusion

The present study noted that both methods have demonstrated high specificity; however, in our experience, OH was significantly more sensitive than 2D TVS for detection of uterine pathologies in patients with RIF and recurrent abortion. It seemed that the OH should be considered as workup method prior to the treatment cycle even in women with normal HSG and/or TVS. 
